# The *SCD5* Gene Modulates Adipogenic Differentiation via the *WNT5B* Signaling Pathway in Xinjiang Brown Cattle

**DOI:** 10.3390/ani15243547

**Published:** 2025-12-10

**Authors:** Yiran Wang, Wanping Ren, Wei Shao, Yuxin Zhou, Yili Liu, Junwei Cao, Fengju Wang, Jingdong Bi, Liang Yang

**Affiliations:** Xinjiang Herbivore Nutrition Laboratory for Meat & Milk, College of Animal Science, Xinjiang Agricultural University, No. 311 Nongda East Road, Urumqi 830052, China; wyr2107937740@163.com (Y.W.); rwp15999154824@163.com (W.R.); dksw@xjau.edu.cn (W.S.); zyxgy1123@163.com (Y.Z.); liuyili6781@163.com (Y.L.); cao250107@163.com (J.C.); fengju0906@163.com (F.W.); 15605444389@163.com (J.B.)

**Keywords:** *SCD5*, WNT5B pathway, preadipocyte, proliferation, adipogenic differentiation

## Abstract

This study investigated the role of *SCD5* in bovine preadipocyte proliferation and adipogenic differentiation. *SCD5* overexpression suppressed proliferation, reducing the percentage of EdU-positive cells and overall cell viability (*p* < 0.05). It also downregulated proliferative factors *CDK1* and *CDK2* (*p* < 0.05), and reduced lipid accumulation (*p* < 0.001) along with key adipogenic markers *PPARγ*, *C/EBPα*, and *FABP4* (*p* < 0.01). Conversely, *SCD5* knockdown promoted these processes. Mechanistically, *SCD5* overexpression downregulated *WNT5B* (*p* < 0.05), while knockdown had the opposite effect. Silencing *WNT5B* significantly decreased *β-catenin* transcription (*p* < 0.05), total protein (*p* < 0.05), and phosphorylation (*p* < 0.001). In conclusion, our findings identify *SCD5* as a novel negative regulator of bovine preadipocyte proliferation and differentiation, which exerts its function through the *WNT5B*/*β-catenin* signaling axis. This discovery elucidates previously uncharacterized regulatory mechanisms underlying adipogenesis in livestock species.

## Simple Summary

The deposition of fat in beef cattle directly impacts meat yield and quality. To better understand the regulation of adipocyte (fat cell) development, this study focused on a gene called *SCD5*. We found that *SCD5* acts as a “brake” in the formation of bovine fat cells: enhancing its activity slowed down the multiplication of fat cell precursors and their ability to store fat, whereas reducing its activity accelerated these processes. Further investigation into the mechanism revealed that *SCD5* exerts this braking effect by suppressing a key signaling pathway known as *WNT5B/β-catenin*. In conclusion, our work identifies *SCD5* as a crucial negative regulator of fat formation in cattle. This discovery provides new insights into the biology of fat deposition in livestock and suggests a novel target for future breeding strategies aimed at improving meat quality and yield.

## 1. Introduction

The intramuscular fat (IMF) content within beef serves as a pivotal factor in determining both its quality and market price. IMF denotes the adipose tissue that accumulates within the muscular structure, predominantly localized within the epimysium, perimysium, and endomysium layers. The deposition of intramuscular fat (IMF) arises from the intricate process of fat metabolism in cattle and is modulated by a multitude of factors, including breed, age, nutritional status, and gender, among which breed emerges as the most pivotal determinant [1]. The identification of key regulators of IMF deposition and the mechanistic understanding of their roles are therefore crucial for molecular breeding strategies aimed at improving beef quality.

The development of omics technologies has accelerated the discovery of genes regulating fat deposition. To identify such genes for intramuscular fat (IMF), we performed RNA sequencing using longissimus dorsi muscle samples from Xinjiang Brown cattle with varying IMF content. These findings identified a robust positive correlation between *SCD5* transcript abundance and intramuscular fat content, supporting its role as a candidate gene influencing this trait [2]. Given its role as the rate-limiting enzyme in monounsaturated fatty acid synthesis, *SCD5* is strategically positioned to directly regulate adipogenesis. *SCD5*, an enzyme termed Δ9-desaturase, governs adipogenesis by catalyzing the rate-limiting step in MUFA synthesis, namely the conversion of its substrates palmitic and stearic acids into their products palmitoleic and oleic acids, respectively [3]. In cattle, *SCD* is expressed in key metabolic tissues—including adipose tissue, muscle, and mammary gland—where it centrally regulates lipid metabolism and deposition. *SCD1* and *SCD5* represent the two primary *SCD* isoforms in cattle. *SCD1* has an established pro-adipogenic role, in contrast to the incompletely understood function of *SCD5* [4,5,6]. While existing evidence suggests the role of *SCD5* may be breed-dependent [7,8], its specific function and regulatory mechanisms during bovine preadipocyte proliferation and differentiation remain incompletely understood.

Rios-Esteves and Resh demonstrated that *SCD* inhibition impairs *WNT/β-catenin* signaling by reducing palmitic acid availability, which is essential for proper pathway transduction [9,10]. The canonical *WNT* signaling cascade, mediated by *β-catenin*, plays a well-established role in suppressing adipocyte differentiation within adipose tissue [11]. The role of the non-canonical ligand *WNT5B* in adipogenesis remains controversial. Some studies report that *WNT5B* inhibits differentiation through *β-catenin*-independent mechanisms [12], In contrast, others propose that it promotes adipogenesis by antagonizing the *β-catenin*-mediated signaling cascade, thereby relieving the suppression of *PPARγ* [13]. We hypothesize that *SCD5* regulates adipogenesis in bovine preadipocytes by modulating *WNT5B* expression. To test this, we conducted experiments using Xinjiang Brown cattle. This study investigates how *SCD5* regulates bovine preadipocyte proliferation and differentiation, with a focus on the *WNT5B*-mediated mechanism. Our findings will identify a novel regulatory axis for fat deposition, providing a genetic basis for molecular breeding strategies to improve meat quality in Xinjiang Brown cattle.

## 2. Materials and Methods

### 2.1. Tissue Sample Collection

Primary adipocytes were isolated from adipose tissue obtained from the longissimus dorsi muscle of Xinjiang Brown cattle at a commercial abattoir. Approximately 500 g of tissue was collected under sterile conditions within 1–2 min post-slaughter. The samples were briefly rinsed with 75% ethanol (Jiangsu Intco Medical Products Co., Ltd., Zhenjiang, China) for 15 s, followed by 2–3 washes in hydrochloric acid-buffered PBS (Cytiva, Marlborough, MA, USA). After rinsing, the tissues were placed in a thermally insulated container with PBS (Cytiva, Marlborough, MA, USA) and promptly transferred to the lab, where cell isolation procedures were immediately initiated.

### 2.2. Cell Isolation, Culture, and Differentiation

Primary bovine preadipocytes were isolated by enzymatic digestion and subsequently expanded in a complete growth medium. The growth medium consisted of DMEM/F12 containing 10% FBS, 100 IU/mL penicillin, and 100 μg/mL streptomycin. Prior to induction, a 2-day period of contact inhibition (day - 2) was established by maintaining cells at full confluence. Adipogenic differentiation was initiated using a differentiation medium comprising 2 μM insulin, 0.5 mM IBMX, 1 μM dexamethasone, and 1 μM rosiglitazone. Following the 6-day induction phase, we introduced a maintenance medium (growth medium supplemented with 1 μg/mL insulin) to support terminal maturation.

### 2.3. Oil Red O Staining

Following 8 days of differentiation, cellular samples underwent three PBS washes and were fixed with 4% PFA (Wuhan Kanos Technology Co., Ltd., Wuhan, China) for 40 min in darkness. After fixation, cells underwent three PBS rinses to visualize lipid deposits through 30 min staining using an Oil Red O working solution (Sigma-Aldrich, St. Louis, MO, USA). Image acquisition for subsequent analysis was performed using an inverted microscope (IX73, Olympus, Tokyo, Japan). Lipid droplets were extracted with isopropanol (Jiangsu Intco Medical Products Co., Ltd., Zhenjiang, China). A microplate reader (Molecular Devices, San Jose, CA, USA) was employed to determine the lipid content based on optical density measurements at 510 nm.

### 2.4. siRNA and Plasmid DNA Transfection

**Figure 1:**
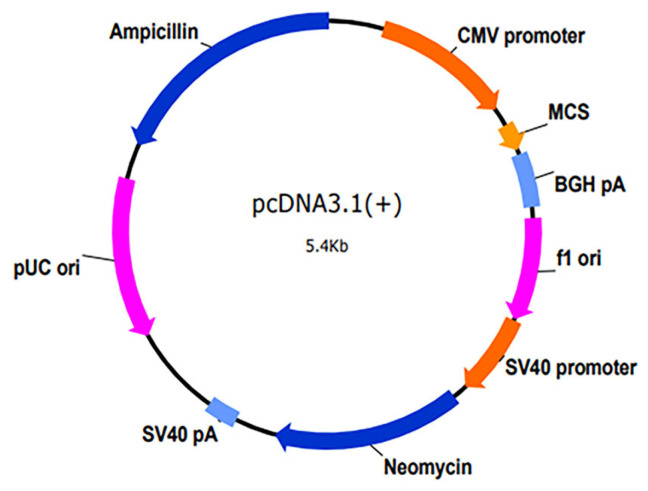
Schematic diagram of the plasmid vector.

Shanghai GenePharma Co., Ltd. (Shanghai, China) supplied all constructs, including the *SCD5* overexpression vector (pcDNA3.1-*SCD5*), the empty control vector pcDNA3.1(+), as well as the gene-specific siRNAs targeting *SCD5* and a negative control siRNA. The schematic structure of the pcDNA3.1(+) vector is illustrated in Figure 1. The *SCD5* coding sequence was subcloned into the pcDNA3.1(+) plasmid via restriction enzyme digestion and ligation at its multiple cloning site. The sequences of siRNAs are shown in Table 1. For all experiments involving transfection in 6-well plates, bovine preadipocytes were seeded and transfected at approximately 70–80% confluence using Lipofectamine 2000 transfection reagent (Invitrogen, Waltham, MA, USA) according to the manufacturer’s instructions. The following standard conditions were applied: the overexpression group and the negative control group received 2.5 μg of the corresponding vector (pcDNA3.1-*SCD5* or empty vector, respectively), whereas the knockdown group and the scramble control group were transfected with 100 μM of specific siRNA-*SCD5* or control siRNA, respectively. After a 48 h transfection period, cells were routinely harvested for subsequent analysis.

### 2.5. Cell Proliferation Test

To determine proliferation rates, we employed the CCK-8 assay (Meilunbio, Shanghai, China) to monitor metabolic activity and the EdU assay (Beyotime Biotechnology, Shanghai, China) to evaluate DNA synthesis. Upon reaching adherence in 96-well plates, preadipocytes were transfected with either the *SCD5* overexpression plasmid or a corresponding siRNA to achieve *SCD5* upregulation or knockdown, respectively. After 48 h, cultures were supplemented with 20 μL CCK-8 reagent and underwent subsequent incubation (37 °C, 5% CO_2_) for 4 h. We measured the absorbance at 450 nm using a microplate reader from Molecular Devices (San Jose, CA, USA). Following seeding in 24-well plates and transfection with the respective plasmid or siRNA, cells were cultured for 48 h and subsequently pulsed with 5-ethynyl-2′-deoxyuridine (EdU) for 2 h prior to detection. Subsequently, Cellular fixation was carried out using a 4% paraformaldehyde solution sourced from Wuhan Canos Technology Co., Ltd. (Wuhan, China) and subjected to the Click-iT reaction by adding the mixture and incubating in the dark for 30 min, as per the manufacturer’s protocol. A 10 min DAPI staining procedure was applied to visualize the cell nuclei. Stained cells were visualized and imaged on an Olympus IX73 inverted microscope (Tokyo, Japan) featuring a U-RFL-T fluorescence illuminator.

### 2.6. RNA Extraction and RT-qPCR

To isolate total RNA, we employed RNAiso Plus reagent (TaKaRa Bio Inc., Dalian, China) on the collected cell samples. its concentration was then quantified on a spectrophotometer (Thermo Scientific, Waltham, MA, USA). The RNA concentration was standardized, and cDNA synthesis was carried out as outlined in the manufacturer’s protocol for the reverse transcription kit (TaKaRa Bio Inc., Dalian, China). Gene expression was quantified by real-time PCR using the SYBR Premix Ex Taq™ kit (TaKaRa Bio) on a Bio-Rad ABI platform. According to the 2^−ΔΔCT^ method, expression levels were normalized to *GAPDH* as the internal control. Each sample was run in technical triplicate. All amplification primers were synthesized by Sangon Biotech (Shanghai, China), with their sequences detailed in Table 2.

### 2.7. Western Blot

On ice, cells were lysed with RIPA reagent for total protein extraction (Beyotime Biotechnology, Shanghai, China). We determined the protein concentration using a BCA protein assay kit (Thermo Fisher Scientific, Waltham, MA, USA), protein extracts were combined with 5× loading buffer and denatured at 100 °C for 10 min in a PCR system (Bio-Rad Laboratories) before electrophoresis. Protein separation was achieved by SDS-PAGE with a 4–12% GenScript ExpressPlus™ gel (GenScript, Piscataway, NJ, USA) at 90 V. The electrophoretic process was discontinued once the pre-stained protein marker (Thermo Fisher Scientific) had migrated to the gel bottom. The “sandwich” wet transfer method was employed to transfer proteins onto a PVDF membrane from Millipore (Billerica, MA, USA). A blocking step was performed by incubating the membrane for one hour at ambient temperature in a solution of 5% non-fat dry milk (Thermo Fisher Scientific, Waltham, MA, USA) prepared in 1× TBST (Solarbio Science & Technology Co., Ltd., Beijing, China). After blocking, the membrane was incubated overnight at 4 °C with the primary antibody prepared in a specialized dilution buffer: GAPDH (1:5000, Proteintech, Wuhan, China, cat #10494-1-AP), CDK1 (1:2000, Proteintech, Wuhan, China, cat #19532-1-AP), CDK6 (1:1000, Proteintech, Wuhan, China, cat #14052-1-AP), PPARγ (1:1000, Proteintech, Wuhan, China, cat #16643-1-AP), C/EBPα (1:1000, Proteintech, Wuhan, China, cat #29388-1-AP), SCD5 (1:1000, Aviva Systems Biology, San Diego, CA, USA, cat #ARP65045_P050), WNT5B (1:1000, biorbyt, Cambridge, UK, cat #orb577989), β-Catenin (1:1000, Abcam, Cambridge, UK, cat #ab32572), and p-β-Catenin (1:500, Affbiotech, Beijing, China, cat #affbiotech). Following three 10 min TBST washes, the antibody binding step was carried out at ambient temperature for 60 min using an HRP-conjugated anti-rabbit secondary antibody (1:1000 dilution, Proteintech, Wuhan, China, catalog #RGAROO4). Three sequential washes, each lasting 10 min, were performed on the membrane. We detected protein bands using an enhanced chemiluminescence (ECL) method with a chemiluminescence reagent from Beyotime Biotechnology (Shanghai, China). The resulting signals were captured using the CHAMPCHEM™ instrument (Sage Creation Science Co., Ltd., Beijing, China), and band intensities were quantified with ImageJ (version 2.14.0/1.54k, National Institutes of Health, Bethesda, MD, USA) software.

### 2.8. Statistical Analysis

Data were organized and preliminarily cleaned using Microsoft Excel (2021, Microsoft Corporation, WA, USA) and subsequently analyzed using SPSS (version 26.0, IBM, Armonk, NY, USA). All data are presented as the mean ± standard deviation (SD). The specific statistical tests applied were as follows: independent-samples *t*-tests were used for comparisons between two groups, and one-way analysis of variance (ANOVA) followed by Duncan’s post hoc test was used for comparisons among multiple groups. Statistical significance was defined as *p* < 0.05 for significant differences and *p* < 0.01 for highly significant differences.

## 3. Results

### 3.1. Expression Profile of SCD5 During Adipogenic Differentiation

**Figure 2:**
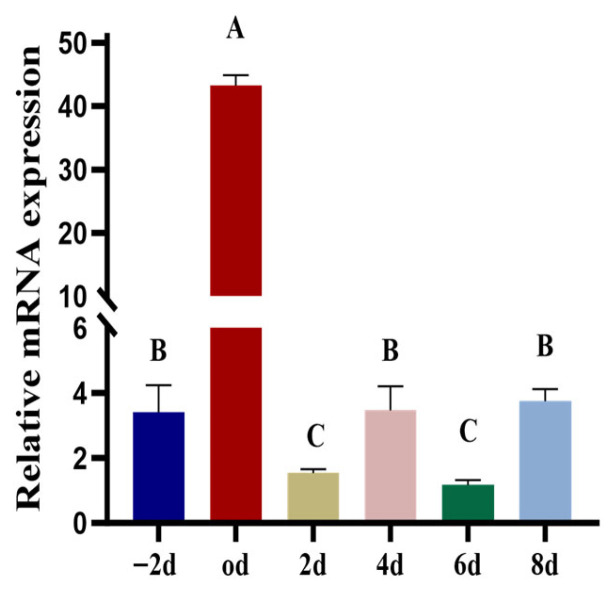
Expression Profile of the *SCD5*. The time points were as follows: day - 2 represents two days before induction, day 0 is the initiation of induction, and day 2–8 represents the post-induction time points. Data are presented as the mean ± standard deviation (*n* = 3). Different uppercase letters indicate a highly significant difference (*p* < 0.01).

As shown in Figure 2, *SCD5* expression peaked at differentiation day 0. While relative elevations were also noted on day - 2, 4, and 8, the Transcript levels on day 2 and 6 were significantly lower, representing the lowest points throughout the observed period (*p* < 0.01).

### 3.2. Overexpression and Knockdown of the SCD5

**Figure 3:**
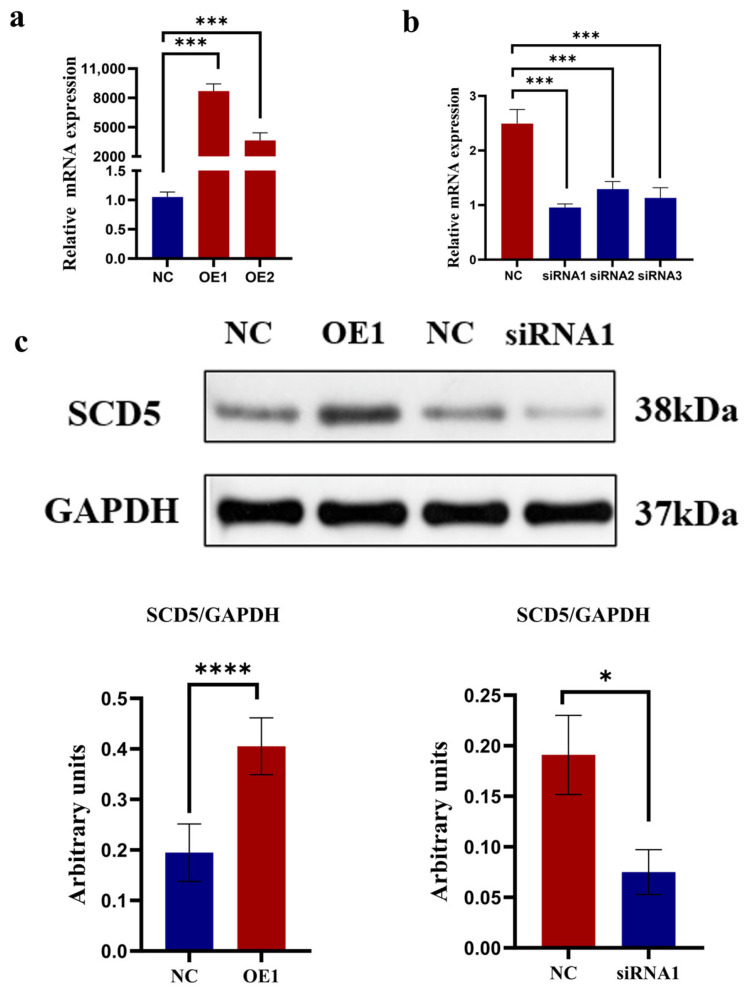
Overexpression and Knockdown of the *SCD5*. (**a**) *SCD5* mRNA levels 48 h after transfection with overexpression plasmids; (**b**) *SCD5* mRNA levels 48 h after transfection with siRNA; (**c**) *SCD5* protein levels 48 h post-transfection. Data represent the mean ± SD from three independent replicates (*n* = 3). Treatments: OE (pcDNA3.1-*SCD5*) at 2.5 μg/well (OE1) and 1.9 μg/well (OE2); siRNA-*SCD5* at 100 μM/well (siRNA1, 2, 3). Statistical significance: * *p* < 0.05, *** *p* < 0.001, **** *p* < 0.0001.

The results are shown in Figure 3. A marked difference in transfection efficiency was observed for the *SCD5* overexpression (OE) group compared to the negative control (NC), and this difference was statistically highly significant (*p* < 0.001). Based on its superior transfection efficiency relative to the OE2 group, the OE1 construct was selected for all further functional experiments targeting *SCD5* (Figure 3a). To identify the most effective siRNA for *SCD5* knockdown, we screened three candidates (siRNA1, 2, and 3). All three siRNAs effectively silenced *SCD5* expression, showing significant suppression (*p* < 0.001) relative to negative control cells, and siRNA1 proved most potent. Consequently, siRNA1 was employed for all subsequent interference trials targeting the *SCD5* (Figure 3b). To further confirm the overexpression and knockdown effects of the *SCD5*, protein samples were collected from bovine preadipocytes 48 h after transfection with OE1 and siRNA1. An upregulation of *SCD5* protein was evident in bovine preadipocytes following OE1 transfection, as determined by Western blot analysis (*p* < 0.0001), while siRNA1 transfection significantly decreased *SCD5* protein abundance (*p* < 0.05) (Figure 3c). The full-length, uncropped blots are provided in Supplementary Figure S1. Collectively, these findings confirm the successful establishment of both *SCD5* gain- and loss-of-function models, which are suitable for subsequent functional characterization.

### 3.3. Overexpression of SCD5 Inhibits the Proliferation of Bovine Preadipocytes

**Figure 4:**
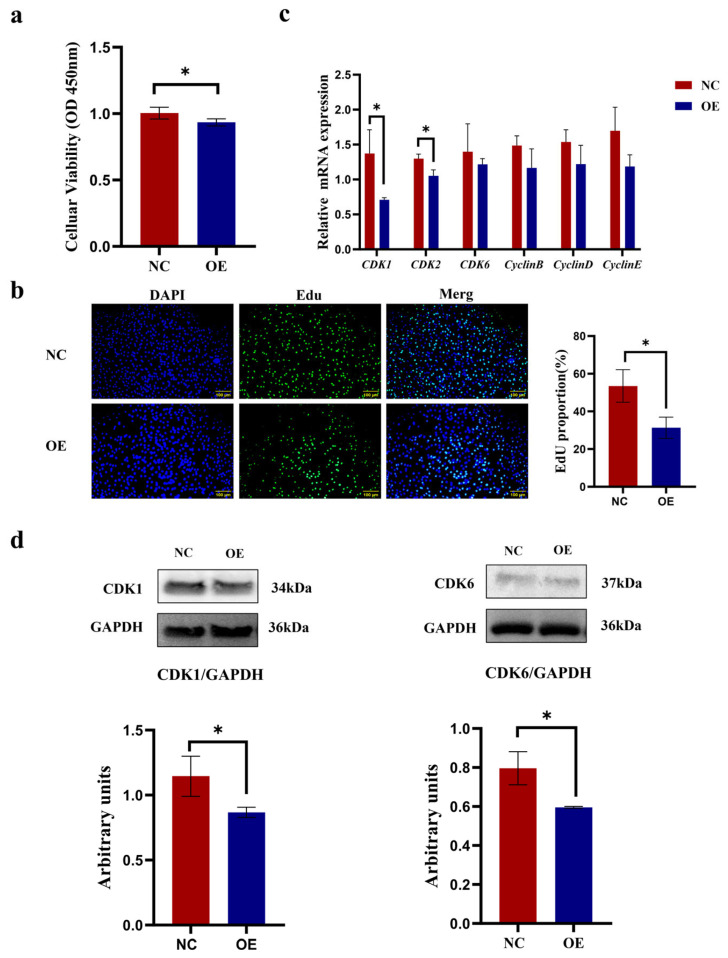
Effect of *SCD5* Overexpression on the Proliferation of Bovine Preadipocytes. (**a**) Cell viability assessed by CCK-8 assay 48 h post-transfection (*n* = 5); (**b**) EdU staining (Scale bar = 100 μm) at 48 h (*n* = 3); (**c**) mRNA levels of proliferation-related genes at 48 h (*n* = 3); (**d**) Protein levels of proliferation-related genes at 48 h (*n* = 3). All data are shown as mean ± SD. The overexpression group (OE) received pcDNA3.1-*SCD5*, compared to a negative control (NC). Asterisks indicate significant differences (* *p* < 0.05).

In the results, as shown in Figure 4, data from both CCK-8 and EdU assays demonstrated that *SCD5* overexpression caused significant suppression of proliferation in bovine preadipocytes compared to control cells (*p* < 0.05, Figure 4a,b). Relative to the negative control, OE treatment significantly suppressed *CDK1* and *CDK2* expression (*p* < 0.05). Although no statistically significant alterations were observed in the mRNA levels of *CDK6*, *CyclinB*, *CyclinD*, and *CyclinE* (*p* > 0.05), a discernible decreasing pattern was noted (Figure 4c). Western blot analysis confirmed a marked downregulation of *CDK1* and *CDK6* levels in *SCD5*-overexpressing cells relative to negative controls (*p* < 0.05, Figure 4d). The full-length, uncropped blots are provided in Supplementary Figure S2. Collectively, these findings demonstrate that *SCD5* overexpression inhibits the proliferative capacity of bovine preadipocytes.

### 3.4. SCD5 Knockdown Promotes Bovine Preadipocyte Proliferation

**Figure 5:**
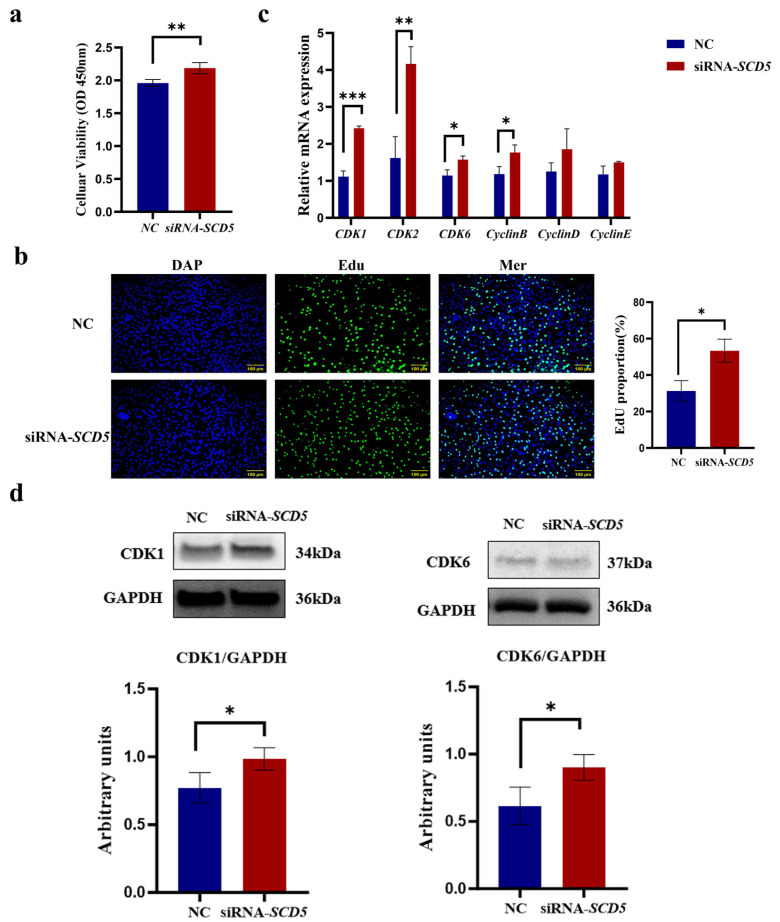
Effect of *SCD5* Interference on the Proliferation of Bovine Preadipocytes. (**a**) Cell viability (CCK-8) 48 h post-transfection (*n* = 6); (**b**) Representative EdU staining (Scale bar = 100 μm) at 48 h (*n* = 3); (**c**) mRNA expression of proliferation-related genes (*n* = 3); (**d**) Protein expression of proliferation-related genes. Values are presented as mean ± SD (*n* = 3 for (**b**–**d**)). Significant differences from the negative control (NC) are marked: * *p* < 0.05, ** *p* < 0.01, *** *p* < 0.001.

As shown in Figure 5, the CCK8 assay demonstrated that the siRNA-*SCD5* treatment significantly promoted the proliferation of bovine preadipocytes (*p* < 0.01, Figure 5a). The EdU assay further validated that the siRNA-*SCD5* treatment significantly enhanced bovine preadipocyte proliferation (*p* < 0.05, Figure 5b). Relative to the negative control, siRNA-*SCD5* significantly elevated the mRNA levels of *CDK1* and *CDK2* (*p* < 0.01), as well as those of *CDK6* and *CyclinB* (*p* < 0.05). However, the expression levels of *CyclinD* and *CyclinE* genes did not show significant differences (*p* > 0.05), although an increasing trend was observed (Figure 5c). *SCD5* knockdown induced a concomitant upregulation of *CDK1* and *CDK6* protein expression, as validated by immunoblot assay (*p* < 0.05, Figure 5d). The full-length, uncropped blots are provided in Supplementary Figure S2. Collectively, these data indicate that *SCD5* knockdown promotes bovine preadipocyte proliferation.

### 3.5. SCD5 Overexpression Suppresses Bovine Preadipocyte Differentiation

**Figure 6:**
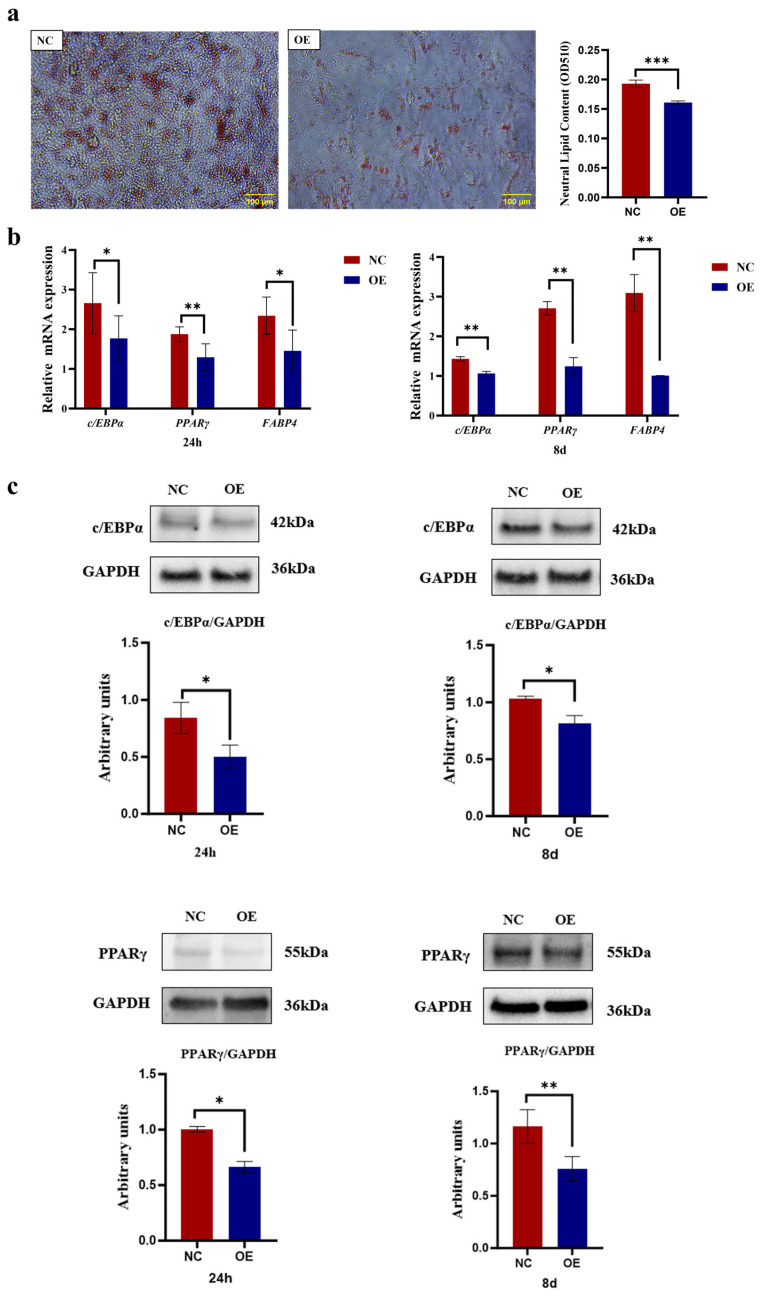
Effect of *SCD5* Overexpression on the Differentiation of Bovine Preadipocytes. (**a**) Lipid accumulation assessed by Oil Red O staining at day 8 of differentiation (Scale bar = 100 μm). The extracted Oil Red O dye was quantified by measuring its absorbance at 510 nm and the OD value represents the quantified intracellular triglyceride (TG) content (*n* = 3); (**b**) mRNA levels of differentiation markers at 24 h and 8 d (*n* = 3); (**c**) Protein levels of differentiation markers at 24 h and 8 d (*n* = 3). All values represent mean ± SD. OE: pcDNA3.1-*SCD5*; NC: negative control. * *p* < 0.05, ** *p* < 0.01, *** *p* < 0.001.

As shown in Figure 6, microscopic observation of the cells showed pronounced red lipid droplets following Oil Red O staining. The OE group exhibited notably fewer lipid deposits compared to the NC group. Upon microscopic examination, it was evident that the NC group possessed greater lipid accumulation compared to the OE group. Quantification of lipid droplets was performed after extraction with isopropanol, with the NC group showing significantly higher lipid content than the OE group (*p* < 0.001, Figure 6a). Compared to the negative control, *SCD5* overexpression induced a marked downregulation of key adipogenic genes at both 24 h and 8 days post-differentiation. Specifically, *PPARγ* (*p* < 0.01), *C/EBPα*, and *FABP4* (*p* < 0.05) were suppressed at 24 h, with a sustained suppression of all three regulators at 8 days (*p* < 0.01, Figure 6b). Similarly, treatment with the *SCD5* plasmid significantly reduced the protein expression levels of *PPARγ* and *C/EBPα* at 24 h post-differentiation (*p* < 0.05), and at the day 8 time point, the protein levels of key adipogenic transcription factors *PPARγ* and *C/EBPα* were also markedly reduced (*p* < 0.01, Figure 6c). The full-length, uncropped blots are provided in Supplementary Figure S3. Collectively, these findings demonstrate that SCD5 overexpression suppresses adipogenesis in bovine preadipocytes.

### 3.6. SCD5 Knockdown Promotes Bovine Preadipocyte Differentiation

**Figure 7:**
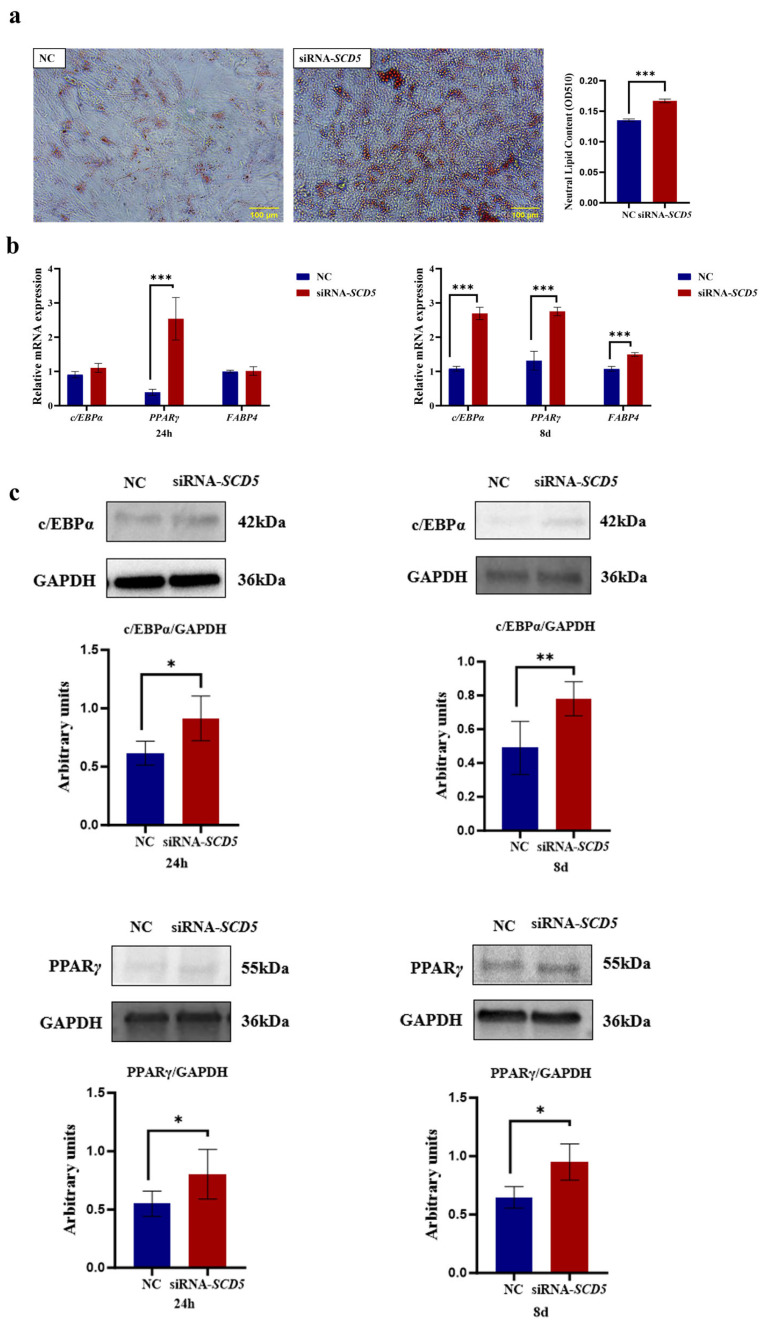
Effect of *SCD5* Knockdown on the Differentiation of Bovine Preadipocytes. (**a**) Lipid accumulation by Oil Red O staining at day 8 of differentiation (Scale bar = 100 μm). Quantification at 510 nm after extraction and the OD value represents the quantified intracellular triglyceride (TG) content (*n* = 3); (**b**) mRNA expression of differentiation-related genes at 24 h and 8 d; (**c**) Protein expression of differentiation-related genes at 24 h and 8 d. Data are presented as mean ± SD (*n* = 3). NC: negative control. * *p* < 0.05, ** *p* < 0.01, *** *p* < 0.001.

As shown in Figure 7, microscopic analysis revealed numerous lipid droplets within cells, as presented After Oil Red O staining, the lipid droplets appeared red, and it was visually apparent that the NC group contained fewer lipid droplets compared to the siRNA group. Lipid quantification was conducted following dye extraction using isopropanol. The siRNA-*SCD5* group exhibited a marked increase in lipid content compared to the negative control (NC) (*p* < 0.001, Figure 7a). At both 24 h and 8 days post-differentiation, *SCD5* knockdown significantly elevated the mRNA levels of key adipogenic genes relative to the control. *PPARγ* was upregulated at the 24 h mark (*p* < 0.001), while *PPARγ*, *C/EBPα*, and *FABP4* all showed elevated expression at day 8 (*p* < 0.001, Figure 7b). Additionally, siRNA*-SCD5* treatment significantly increased the protein levels of *PPARγ* and *C/EBPα* at 24 h post-differentiation (*p* < 0.05); at 8 days, the levels of both *PPARγ* (*p* < 0.05) and *C/EBPα* (*p* < 0.01) remained significantly elevated (Figure 7c). The full-length, uncropped blots are provided in Supplementary Figure S3. In summary, our results demonstrate that *SCD5* knockdown promotes adipogenic differentiation in bovine preadipocytes.

### 3.7. Effect of the SCD5 on the WNT Signaling Pathway

**Figure 8:**
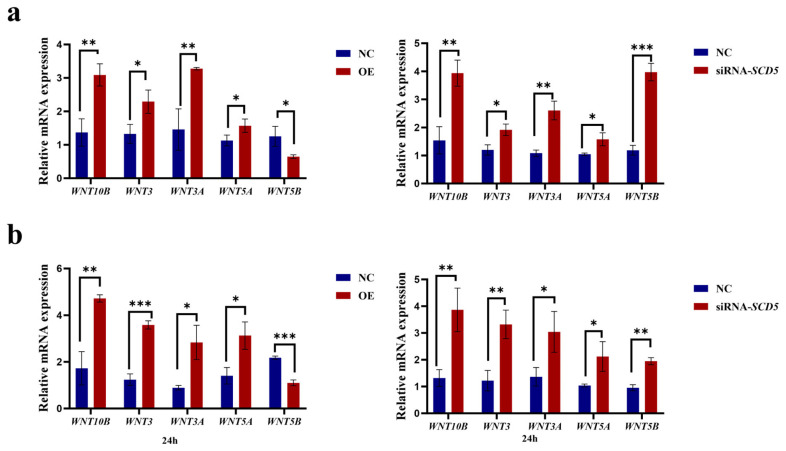
Effect of the *SCD5* on the *WNT* Signaling Pathway. (**a**) mRNA expression of *WNT* genes 48 h post-transfection; (**b**) mRNA expression of *WNT* genes 24 h after differentiation induction. Values are shown as mean ± SD (*n* = 3). OE: pcDNA3.1-*SCD5*; NC: negative control. * *p* < 0.05, ** *p* < 0.01, *** *p* < 0.001.

As demonstrated in Figure 8, relative to the negative control group, overexpression of *SCD5* significantly downregulated *WNT5B* (*p* < 0.05), but upregulated *WNT10B* and *WNT3A* (*p* < 0.01), as well as *WNT3* and *WNT3A* (*p* < 0.05). After interference with siRNA-*SCD5*, all tested WNTs (*WNT10B*, *WNT5B*, *WNT5A*, *WNT3*, and *WNT3A*) were significantly upregulated, with *WNT10B/3A* (*p* < 0.01), *WNT5A/3* (*p* < 0.05), and *WNT5B* (*p* < 0.001) showing the most significant increases (Figure 8a). Compared to the negative control group, overexpression of *SCD5* significantly downregulated *WNT5B* (*p* < 0.001), but upregulated *WNT10B* (*p* < 0.01), *WNT3* (*p* < 0.001), and *WNT3A* and *WNT5A* (*p* < 0.05). After interference with siRNA-*SCD5*, all tested WNTs (*WNT10B*, *WNT5B*, *WNT5A*, *WNT3*, and *WNT3A*) were significantly upregulated, with *WNT10B/3/5B* (*p* < 0.01) and *WNT3* and *WNT5A* (*p* < 0.05) showing significant increases (Figure 8b). Our findings support a bifunctional regulation of the *WNT* pathway by *SCD5*, supported by a compelling inverse correlation with *WNT5B* expression. The persistence of this relationship suggests that the downregulation of *WNT5B* is functionally integral to the inhibitory mechanism orchestrated by *SCD5*.

### 3.8. SCD5 Regulates Adipogenesis Through the WNT5B Signaling Pathway

**Figure 9:**
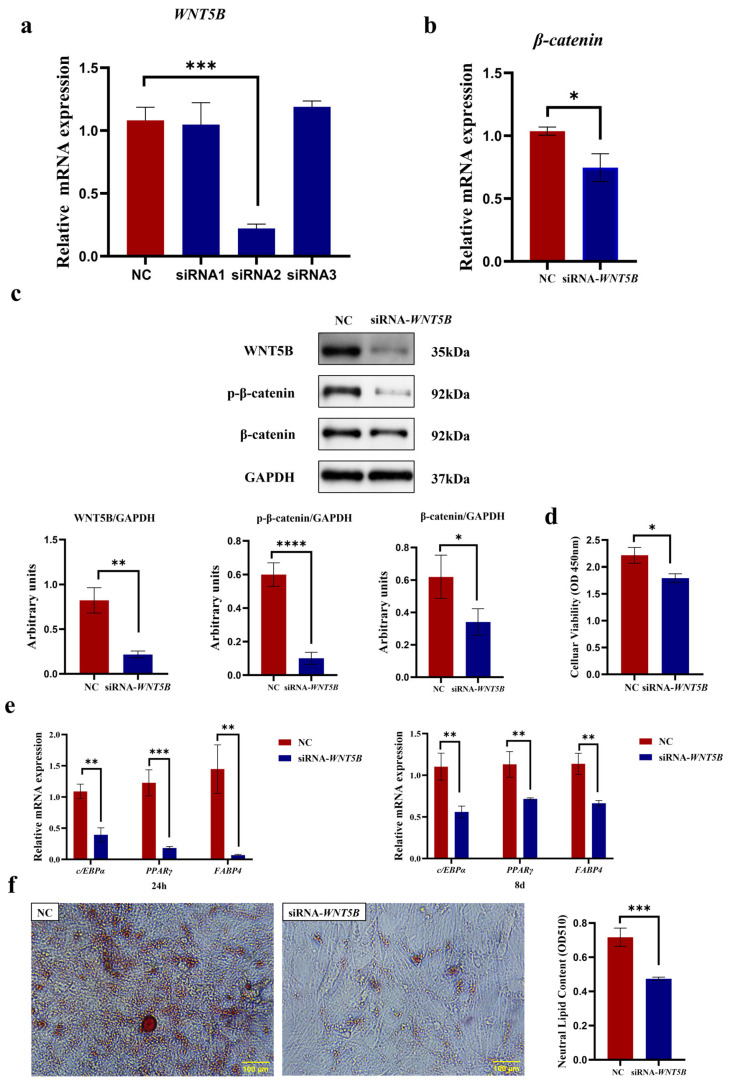
The *SCD5* Regulates Adipogenesis Through the *WNT5B* Signaling Pathway. (**a**) *WNT5B* interference efficiency 48 h post-transfection. siRNA2 was selected for subsequent experiments (*n* = 3). (**b**) *β-catenin* mRNA expression after *WNT5B* silencing (*n* = 3). (**c**) Protein levels of *WNT5B*, *β-catenin*, and phosphorylated *β-catenin* (*p-β-catenin*) as determined by Western blot 48 h post-transfection (*n* = 3). (**d**) Cell proliferation (CCK-8) 48 h post-transfection (*n* = 3). (**e**) mRNA expression of differentiation markers at 24 h and 8 d. (**f**) Lipid accumulation by Oil Red O staining at day 8. Quantification at 510 nm after extraction and the OD value represents the quantified intracellular triglyceride (TG) content. All data are expressed as mean ± SD. * *p* < 0.05, ** *p* < 0.01, *** *p* < 0.001, **** *p* < 0.0001.

As shown in Figure 9, knockdown of *WNT5B* not only effectively suppressed its own transcript abundance (*p* < 0.001) but also consequently reduced the mRNA expression of *β-catenin*, a key downstream mediator of the canonical *WNT* pathway (*p* < 0.05, Figure 9a,b). *β-catenin* protein levels were also significantly reduced (*p* < 0.01), with a concomitant decrease in phosphorylated *β-catenin* (*p* < 0.0001, Figure 9c). The full-length, uncropped blots are provided in Supplementary Figure S4. CCK-8 assay results demonstrated that siRNA-*WNT5B* treatment significantly inhibited the proliferation of bovine preadipocytes (*p* < 0.05, Figure 9d). Compared to the negative control group, *WNT5B* knockdown significantly reduced the expression of *PPARγ* (*p* < 0.001), *C/EBPα*, and *FABP4* genes at 24 h post-differentiation and at 8 days post-differentiation (*p* < 0.01, Figure 9e). Large lipid droplets were visible within the cells, and lipid droplets were vividly stained red by Oil Red O, allowing for a visual comparison of lipid content. The NC group exhibited more lipid droplets than the siRNA-treated group. Quantification of lipid droplets, after extraction with isopropanol, showed a highly significant difference between the NC and siRNA groups (*p* < 0.001, Figure 9f). These findings reveal a promotional role for *WNT5B* in bovine preadipocyte proliferation and differentiation, while *SCD5* suppresses this process through transcriptional downregulation of *WNT5B*.

## 4. Discussion

### 4.1. Expression and Functional Study of the SCD5 in Adipogenic Differentiation

The present study carried out an in-depth and comprehensive exploration of the expression dynamics and functional mechanisms of the *SCD5* across the entire process of adipogenic differentiation. The trial results showed that *SCD5* expression changed dynamically during the 8-day adipogenic differentiation. mRNA levels fluctuated substantially, and were significantly elevated at key time points: at the onset (day 0), midpoint (day 4), and termination (day 8) of the process. We detected a significant rise in *SCD5* expression at the onset of differentiation (day 0). A plausible explanation for this initial increase is its direct response to the immediate activation of core adipogenic regulators, *PPARγ* and *C/EBPα*. This interpretation is supported by an established mechanistic pathway: *PPARγ* enhances *FXR* expression, which then forms a positive feedback loop to amplify *SCD5* expression in a *PPARγ*-dependent manner, thereby promoting adipogenesis and cellular differentiation [14]. Furthermore, *EGR2* and *SREBP1a* have been demonstrated to directly activate the bovine *SCD5* promoter. This direct transcriptional regulation provides a mechanistic basis for the rapid increase in *SCD5* expression during early differentiation [15]. At day 2, the expression level decreased. This decline could be indicative of an active transcriptional repression mechanism that fine-tunes the differentiation process. Consistent with this notion, growth hormone (bGH) and growth hormone-releasing factor (bGHR) have been reported to strongly suppress the *SCD* mRNA level in bovine adipose tissue [16]. Expression increased again by the mid-differentiation stage (day 4), a critical period marked by extensive lipid droplet formation. This resurgence likely supports the elevated demand for triglyceride (TG) synthesis and lipid accumulation. This role is corroborated by prior findings that *SCD* regulates both TG synthesis and fatty acid (FA) content in bovine subcutaneous adipose tissue (SAT) [17]. By the late differentiation stage (day 6), expression declined once more, possibly because sufficient lipid accumulation (e.g., oleic acid, palmitic acid, and other *SCD*-derived products) may have been attained. The homeostatic control of lipid synthesis may be achieved through this potential feedback inhibition. In support of this, Keating et al. confirmed that oleic acid, the primary product of the *SCD*-catalyzed reaction, directly suppresses *SCD* promoter activity in dairy cattle, providing a mechanistic basis for this regulatory loop [18]. A final upregulation was observed at the terminal stage (day 8), consistent with the established role of *SCD* as a marker of mature adipocyte differentiation. This is reinforced by studies confirming that *SCD* expression is significantly induced during adipogenesis and acts as a pivotal indicator of its completion [19]. In conclusion, the dynamic expression profile of *SCD5* collectively describes a role that extends beyond that of a metabolic enzyme to a functional coordinator of the transition from proliferation to differentiation in precursor adipocytes. These findings implicate *SCD5* as a promising genetic target for improving intramuscular fat (IMF) deposition, offering a viable strategy to enhance meat quality in livestock.

### 4.2. The SCD5 Suppresses the Proliferation of Bovine Preadipocytes

In beef cattle production, balancing lean growth with intramuscular adiposity (marbling) is a central challenge due to its direct impact on meat quality and value. Intramuscular preadipocyte proliferation represents an early-stage in marbling development, yet its regulatory mechanisms remain incompletely characterized [20]. This study aims to elucidate the specific role of Stearoyl-CoA Desaturase 5 (*SCD5*) during this foundational stage of adipogenesis in cattle. The capacity for fat deposition is primarily governed by the size of the adipocyte pool, which is determined by the proliferation of preadipocytes. The proliferation of adipocytes is a process governed by the cell cycle. This cycle consists of a precisely ordered series of stages, encompassing the S phase for DNA replication, the M phase for cellular division, and the intervening gap phases G1 and G2 [21,22]. As pivotal controllers of cell cycle dynamics, *CDK1* and *CDK2* orchestrate key phase transitions, particularly at the G1/S and G2/M checkpoints [23,24]. *SCD5* overexpression significantly suppressed the proliferation of bovine preadipocytes, as indicated by a decrease in the percentage of EdU-positive cells and reduced CCK-8 activity. This anti-proliferative effect correlated with downregulation of *CDK1* and *CDK2* mRNA and decreased *CDK1* and *CDK6* protein levels. Conversely, *SCD5* knockdown enhanced preadipocyte proliferation, as evidenced by increased EdU-positive cells and CCK-8 values. This pro-proliferative phenotype was consistent with the concomitant upregulation of key cell cycle genes (*CDK1*, *CDK2*, *CDK6*, and *Cyclin B*) at the mRNA level, as well as elevated *CDK1* and *CDK6* protein levels. Collectively, our findings define a role for *SCD5* that extends beyond lipid metabolism, establishing it as a direct regulator of cell proliferation. Previous studies have reported that *SCD5* suppresses proliferation in renal clear cell carcinoma, demonstrating its conserved anti-proliferative function [25]. This finding is further supported by Zhao et al., who showed that *SCD5* negatively regulates the cell cycle [26]. Collectively, these two findings provide robust support for the results of this research trial. *SCD5* catalyzes the biosynthesis of monounsaturated fatty acids, notably oleic acid. This same fatty acid has been demonstrated to inhibit tumor cell proliferation (Menendez et al., 2005) [27], suggesting a potential mechanistic link through which *SCD5* may influence cell growth. Our findings suggest that *SCD5* and *SCD1* may function independently during adipogenesis: whereas *SCD1* enhances lipid storage, *SCD5*-derived oleic acid could act as a signaling molecule to limit cell proliferation. This observation aligns with the established mechanism wherein *SCD1*-derived oleic and palmitoleic acids serve as endogenous ligands for *PPARγ*. Their binding activates this master transcriptional regulator of adipogenesis, leading to the direct upregulation of genes responsible for lipid deposition [28]. Previous studies establish that oleic acid acts as a ligand for *PPARγ*, a central regulator of adipose tissue development and related gene expression [29,30]. Furthermore, activation of *PPARγ* by its ligands can induce cell cycle arrest [31]. Our findings collectively propose a testable hypothesis: oleic acid, a product of *SCD5*, might function as an endogenous ligand for *PPARγ* in bovine preadipocytes, and its activation could thereby trigger cell cycle arrest and suppress preadipocyte proliferation.

### 4.3. The SCD5 Inhibits the Differentiation of Bovine Preadipocytes

This study investigated the regulatory function of *SCD5* in bovine preadipocyte differentiation (adipogenesis)—the process by which these cells develop into mature adipocytes, crucial for adipose tissue development. Oil Red O staining revealed that *SCD5* overexpression suppressed lipid accumulation, whereas its knockdown promoted this process. Consistent with these observations, we subsequently quantified the expression of key adipogenic master regulators to elucidate the molecular basis for this phenotypic shift. *SCD5* overexpression downregulated the mRNA expression of *PPARγ*, *C/EBPα*, and *FABP4*, with corresponding suppression of *PPARγ* and *C/EBPα* at the protein level. Conversely, *SCD5* knockdown consistently enhanced the expression of these genes. Collectively, our results define *SCD5* as an inhibitor of bovine preadipocyte differentiation; whereas its knockdown accelerates adipogenesis. The promotion of bovine preadipocyte differentiation following *SCD5* knockdown is consistent with reports in mouse 3T3-L1 cells [32,33]. Inhibition of tumor cell differentiation by *SCD5* expression has been documented in advanced melanoma [34]. Previous studies establish that *SCD*-derived monounsaturated fatty acids (MUFAs) influence diverse cellular processes, including growth, survival, and differentiation. Notably, the *SCD* product palmitoleic acid is a recognized modulator of WNT signaling [35]. In contrast to the well-understood role of *SCD1* in adipogenesis, the physiological function of *SCD5* in ruminants remains limited, despite both isoforms being present in cattle. Our findings lead us to speculate that *SCD5* might exert its inhibitory effect by possibly modulating the *Wnt/β-catenin* pathway, potentially through alterations in MUFA synthesis. This presents a testable model for future research. Canonical *WNT* signaling activation inhibits adipocyte differentiation through transcriptional suppression of the master adipogenic transcription factors *PPARγ* and *C/EBPα*. The physiological relevance of this inhibitory mechanism in cattle is demonstrated by its documented role in regulating intramuscular fat (IMF) content in the longissimus dorsi. Conversely, *β-catenin* downregulation enhances *PPARγ* expression, thereby promoting bone marrow-derived mesenchymal stem cells to undergo adipogenic differentiation [36]. We therefore propose a testable hypothesis: SCD5 inhibits adipogenesis primarily by modulating the *Wnt/β-catenin* pathway, potentially through alterations in cellular MUFA composition.

### 4.4. SCD5 Regulates Adipogenic Differentiation of Bovine Preadipocytes Through the WNT5B

This study focuses on the interaction between *WNT* signaling and *SCD5* during adipogenesis in bovine preadipocytes. The *WNT* signaling pathway serves as a pivotal negative regulator in the process of adipogenesis, exerting a potent inhibitory effect on adipocyte differentiation through the suppression of lipid synthesis and storage mechanisms [37,38]. Our data show that *SCD5* overexpression suppressed *WNT5B* expression, while its knockdown conversely elevated *WNT5B* levels. Based on their coordinated expression, we propose *WNT5B* as a downstream effector of *SCD5*. Additionally, *WNT* signaling pathway-related proteins such as *Frizzled*, *GSK3β*, and Axin have been established as regulators of fat deposition and adipocyte differentiation [13,38,39]. To investigate the mechanism of *WNT5B,* we silenced its expression in bovine preadipocytes. This attenuation suppressed proliferation, lipid accumulation, and the expression of key adipogenic markers (*PPARγ*, *C/EBPα*, and *FABP4*). This loss-of-function also reduced *β*-*catenin* mRNA, total protein, and phosphorylated protein levels, positioning *WNT5B* upstream of *β-catenin* regulation. Previous studies have shown that *WNT5B* promotes adipogenesis by inhibiting the *β-catenin*-dependent canonical *WNT* signaling pathway [40]. We hypothesize that silencing *WNT5B* relieves its inhibition of the canonical *WNT* pathway, leading to its excessive activation, which may trigger a strong negative feedback mechanism, ultimately resulting in a significant downregulation of *β-catenin*. Consistent with our findings, other studies have reported that *WNT5B* promotes adipogenesis [41]. Furthermore, demonstrated that *WNT5B*, as a ligand for *ROR2*, cooperates with *ROR2* to promote the proliferation of skeletal muscle-derived mesenchymal progenitor cells (MPs) [42], leading to the accumulation of intramuscular adipose tissue. Additionally, directly confirmed that overexpression of *WNT5B* is sufficient to promote adipogenesis [40]. Previous studies have shown that the product of *SCD* catalysis, palmitoleic acid, binds to *WNT* proteins and activates the *WNT* signaling pathway. Inhibiting the phosphorylation of *GSK3β* prevents the degradation of *β-catenin*, leading to its accumulation in the nucleus [9,43]. In summation, the present study unequivocally identifies *SCD5* as a pivotal negative regulator of adipogenesis in bovine preadipocytes. *SCD5* exerts its anti-adipogenic effect by specifically suppressing *WNT5B* transcription, thereby providing direct evidence for the pro-adipogenic role of *WNT5B* [40,42]. This study provides evidence of an upstream regulatory association between *SCD5* and *WNT5B*, a relationship not previously reported in this context. Our findings suggest a previously uncharacterized mechanism through which lipid metabolism enzymes may influence key developmental signaling pathways. These insights contribute novel theoretical perspectives and identify potential targets for metabolic interventions aimed at regulating fat deposition. Furthermore, while the present in vitro findings elucidate a precise mechanistic pathway, the physiological relevance of the *SCD5*-*WNT5B* axis necessitates future investigation in in vivo models to account for systemic metabolic interactions. The confirmation of this pathway in vivo would significantly strengthen its candidacy as a potential biomarker for genetic selection aimed at improving meat quality (e.g., intramuscular fat content) and modulating fat deposition patterns in agriculturally important species. The conservation of these signaling components across mammalian species suggests that our findings may extend beyond bovine biology, offering a paradigm for understanding adipogenesis in other livestock and potentially informing research into human metabolic diseases.

## 5. Conclusions

Our study demonstrates that *SCD5* suppresses both the proliferation and adipogenic differentiation of bovine preadipocytes. Herein, we present compelling first evidence that *SCD5* negatively regulates *WNT5B* expression. This regulatory event subsequently influences the protein stability of *β-catenin*, ultimately leading to the suppression of the adipogenic differentiation process. This discovery significantly deepens our comprehension of the functional attributes of the *SCD* protein family. Moreover, it offers novel experimental evidence that is instrumental in elucidating the distinctive regulatory mechanisms governing the *WNT* signaling pathway during adipose deposition in ruminants.

## Figures and Tables

**Table 1:** siRNA sequences.

	Item	Sense (5′ to 3′)	Antisense (5′ to 3′)
*SCD5*	siRNA1	ACUCCAUGGCUUUCCAGAATT	UUCUGGAAAGCCAUGGAGUTT
	siRNA2	GGUCCGGUUCCAGAGAAAGTT	CUUUCUCUGGAACCGGACCTT
	siRNA3	GUCGCUCACAUGUACGGAATT	UUCCGUACAUGUGAGCGACTT
*WNT5B*	siRNA1	CUCGCCUUGCUGUUCGCCUTT	AGGCGAACAGCAAGGCGAGTT
	siRNA2	UCCGUCUUUGGGAGAGUCCTT	GGACUCUCCCAAAGACGGATT
	siRNA3	GGGCUGUGUACAAGACGGCTT	GCCGUCUUGUACACAGCCCTT
	NC	UUCUCCGAACGUGUCACGUTT	ACGUGACACGUUCGGAGAATT

NC, negative control.

**Table 2:** RT-qPCR Primers.

Gene	mRNA RefSeq ID	Primer Sequence (5′ to 3′)	Product Size
*SCD5*	NM_001076945.1	F: TGGGTGCCATTGGTGAAGGT	124 bp
		R: CCCAGCCAACACATGAAGTC	
*PPARγ*	NM_181024.2	F: TGGAGACCGCCCAGGTTTGC	111 bp
		R: AGCTGGGAGGACTCGGGGTG	
*C/EBPα*	NM_176784.2	F: TGGGCAAGAGCCGGGACAAG	166 bp
		R: ACCAGGGAGCTCTCGGGCAG	
*FABP4*	NM_174314.2	F: TCCTTCAAATTGGGCCAGGAA	218 bp
		R: CCCTTGGCTTATGCTCTCTCA	
*CDK1*	NM_174016.2	F: GTGGAAACCAGGAAGCTTAGC	201 bp
		R: TGCTCTTGACACAACACAGGGA	
*CDK2*	NM_001014934.1	F: GGCATTCCTCTTCCGCTCAT	142 bp
		R: CTGCTAGCTTGATGGACCCA	
*CDK6*	NM_001192301.2	F: GGAGTGCCCACTGAAACCAT	131 bp
		R: ATTTGTCCACTGCTGGTCACC	
*CyclinB*	NM_001045872.1	F: ACACCTACACCAAGTTTCAAATCA	182 bp
		R: ATCGTAGTCCAGCATAGTTAGTTCC	
*CyclinD*	NM_001046273.2	F: ATGAAGGAGACCATCCCCCT	123 bp
		R: CGCCAGGTTCCACTTGAGTT	
*CyclinE*	NM_001192776.1	F: CCTCCAAAGTTGCACCAGTT	195 bp
		R: AGGATACTGAGGCAGGAGCA	
*WNT10B*	XM_010805029.4	F: GTCTCCTGTTCCTGGCGTTGTG	103 bp
		R: CACACGGTGTTGGCGGTCAG	
*WNT3*	XM_005220917.5	F: CTGGGAACGGGTGAAGTGTGTG	120 bp
		R: GCGTCTGGCAAGAGTCCTGATTC	
*WNT3A*	XM_024995392.2	F: AGTTCGGCGGGATGGTGTCTC	123 bp
		R: GGTGCATGTGACTGGCGATGG	
*WNT5A*	XM_005222857.5	F: TCGGATCGCTAGGTCACACTCTC	110 bp
		R: CATTCGCTGGGTCGGACACTTG	
*WNT5B*	XM_059886227.1	F: AGCCAGTAGCCACTCAAGACACC	144 bp
		R: AGCAGGAGACAGGACCACAGATG	
*GAPDH*	NM_001034034.2	F: TGCCCGTTCGACAGATAGCC	148 bp
		R: GCGACGATGTCCACTTTGCC	

## Data Availability

None of the data were deposited in an official repository. The data are available from the corresponding author upon reasonable request.

## Supplementary Materials

The following supporting information can be downloaded at https://www.mdpi.com/article/10.3390/ani15243547/s1. Figure S1: Full-length blot for *SCD5* (corresponding to Figure 3c); Figure S2: Full-length blot for *CDK1* and *CDK6* (corresponding to Figure 4d and Figure 5d); Figure S3: Full-length blots for *PPARγ* and *C/EBPα* (corresponding to Figure 6c and Figure 7c); Figure S4: Full-length blot for *β-catenin*, *p-β-catenin*, and *WNT5B* (corresponding to Figure 9c).

## Abbreviations

The following abbreviations are used in this manuscript:

*β-catenin*	Beta-Catenin
bGH	Bovine Growth Hormone
C16:0	Palmitic Acid
C16:1n-7	Palmitoleic Acid
C18:0	Stearic Acid
C18:1n-9	Oleic Acid
CCK-8	Cell Counting Kit-8
*CDK1/2/6*	Cyclin-Dependent Kinase 1/2/6
*C/EBPα*	CCAAT/Enhancer-Binding Protein Alpha
DMEM/F12	Dulbecco’s Modified Eagle Medium/Nutrient Mixture F-12
EdU	5-Ethynyl-2′-deoxyuridine
*EGR2*	Early Growth Response Protein 2
FA	Fatty Acid
*FABP4*	Fatty Acid-Binding Protein 4
FBS	Fetal Bovine Serum
*FXR*	Farnesoid X Receptor
*GAPDH*	Glyceraldehyde-3-Phosphate Dehydrogenase
IBMX	3-Isobutyl-1-methylxanthine
IMF	Intramuscular Fat
PBS	Phosphate-Buffered Saline
PFA	Paraformaldehyde
*PPARγ*	Peroxisome Proliferator-Activated Receptor Gamma
RT-qPCR	Quantitative Reverse Transcription Polymerase Chain Reaction
SAT	Subcutaneous Adipose Tissue
*SCD*	Stearoyl-CoA Desaturase
*SCD1*	Stearoyl-CoA Desaturase 1
*SCD5*	Stearoyl-CoA Desaturase 5
*SREBP1a*	Sterol Regulatory Element-Binding Transcription Factor 1a
TG	Triglyceride
*Wnt*	Wingless-type MMTV integration site family
*WNT3/3A/5A/5B/10B*	Wnt Family Member 3/3A/5A/5B/10B
