# Supplementary material for: The SCD5 Gene Modulates Adipogenic Differentiation via the WNT5B Signaling Pathway in Xinjiang Brown Cattle
Source: Animals (Basel). 2025 Dec 10;15(24):3547. doi: 10.3390/ani15243547 (PMC12729747; doi:10.3390/ani15243547)

Supplementary Figure S1. Full-length Western blot for SCD5 (corresponding to Fig. 3c).

The lane order is as follows: Lane 1: Prestained protein ladder; Lane 2: pcDNA3.1-NC; Lane 3: pcDNA3.1-SCD5; Lane 4: siRNA-NC; Lane 5: siRNA-SCD5.

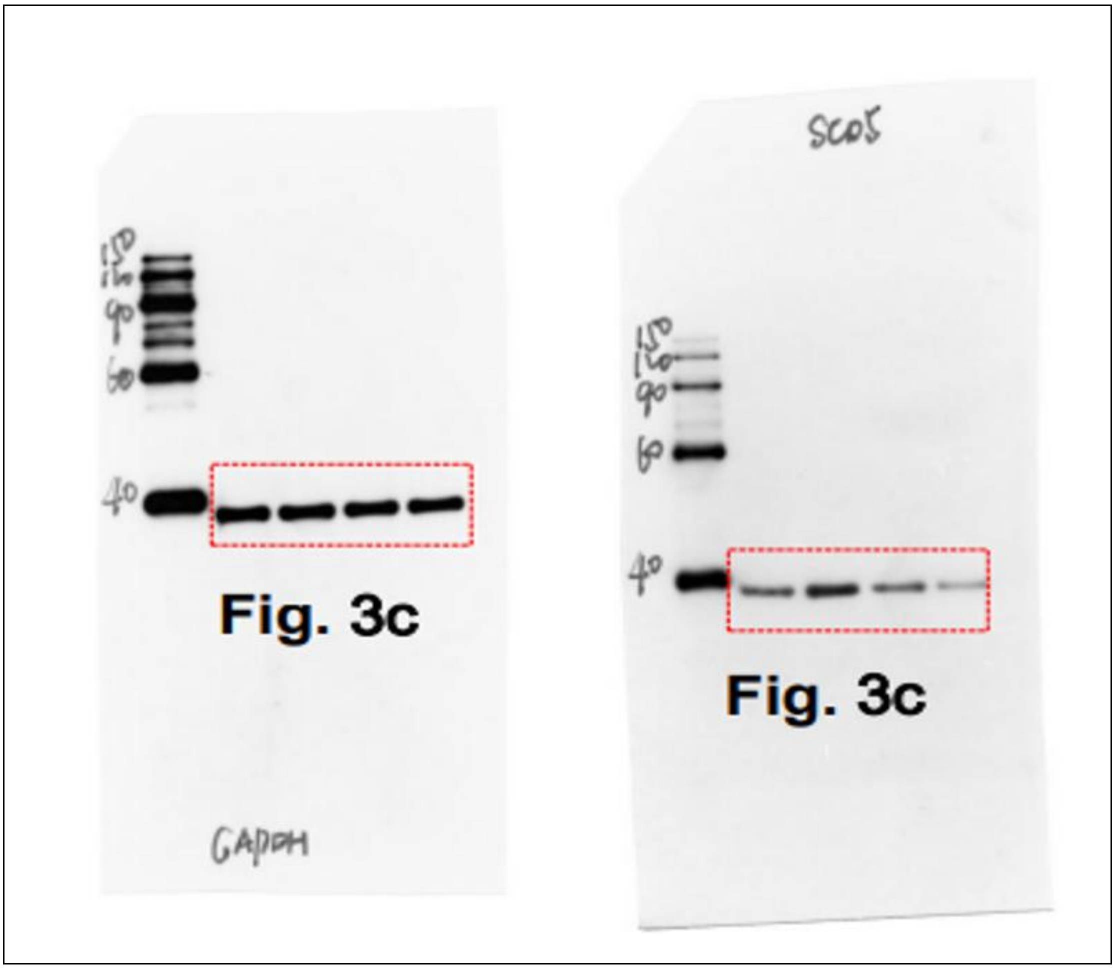

Supplementary Figure S2. Full-length blots for CDK1 and CDK6 (corresponding to Fig. 4d and Fig. 5d).

(a) Full, uncropped membrane probed for CDK1. The lane order is as follows: Lane 1: Prestained protein ladder; Lane 2: pcDNA3.1-NC; Lane 3: pcDNA3.1-SCD5; Lane 4: siRNA-NC; Lane 5: siRNA-SCD5; Lane 6: Prestained protein ladder. The red dashed boxes indicate the lane groupings that were cropped for presentation in Fig. 4d (Lanes 2-3) and Fig. 5d (Lanes 4-5).

(b) Full, uncropped membrane probed for CDK6. The lane order is identical to that shown in (a). The data presented in Fig. 4d and Fig. 5d were cropped from the same lane groupings as outlined for CDK1 in (a).

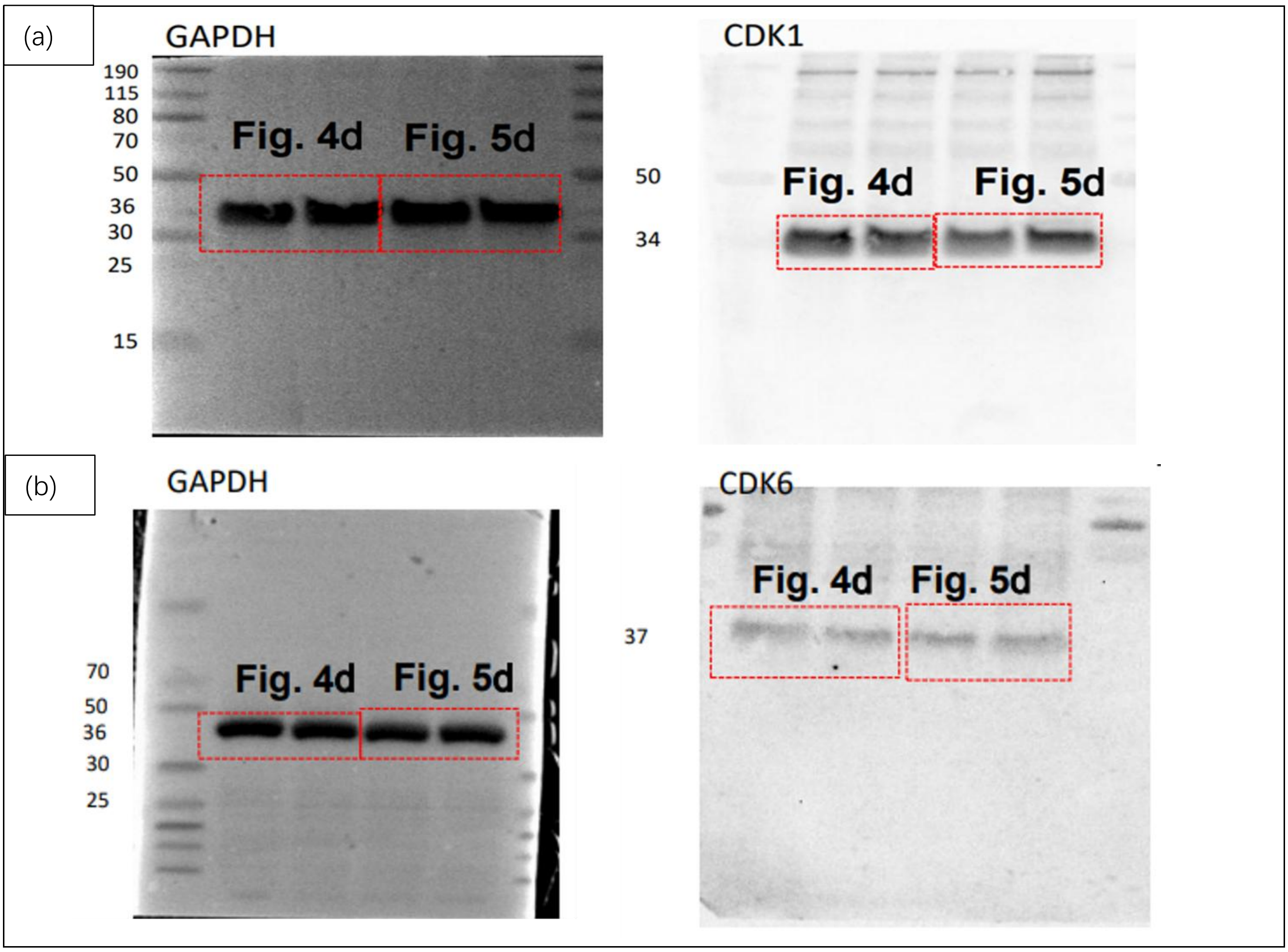

Supplementary Figure S3. Full-length blots for C/EBPα and PPARγ (corresponding to Fig. 6c and Fig. 7c).

Shown are the full, uncropped blots for C/EBPα and PPARγ. The lane order for both blots is as follows: 1, Prestained protein ladder; 2, pcDNA3.1-NC; 3, pcDNA3.1-SCD5; 4, siRNA-NC; 5, siRNA-SCD5; 6, Prestained protein ladder. The red dashed boxes indicate the lane groups cropped for presentation in Fig. 6c (Lanes 2-3) and Fig. 7c (Lanes 4-5). The data presented in the main figures for each protein were cropped from these corresponding regions.

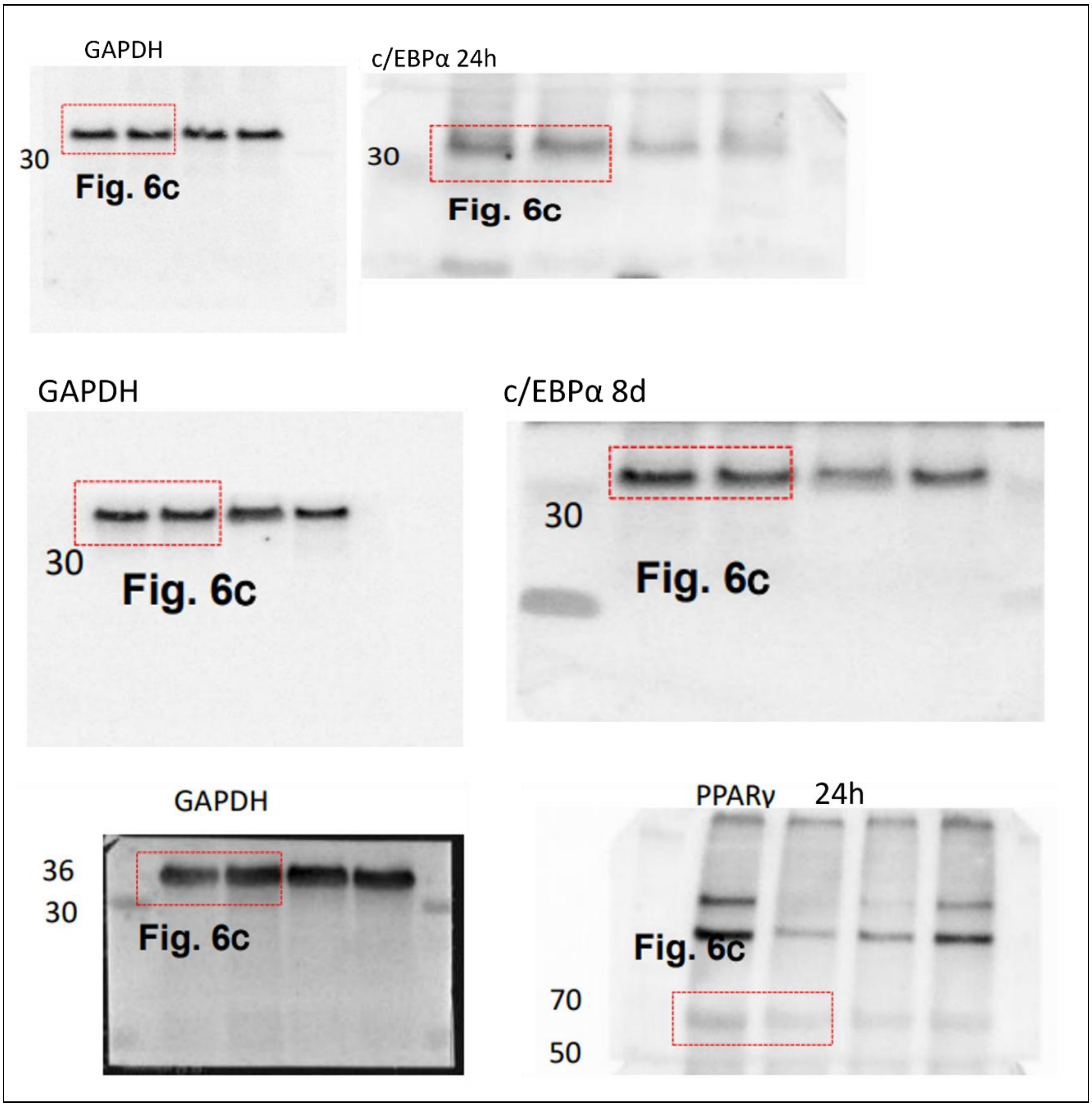

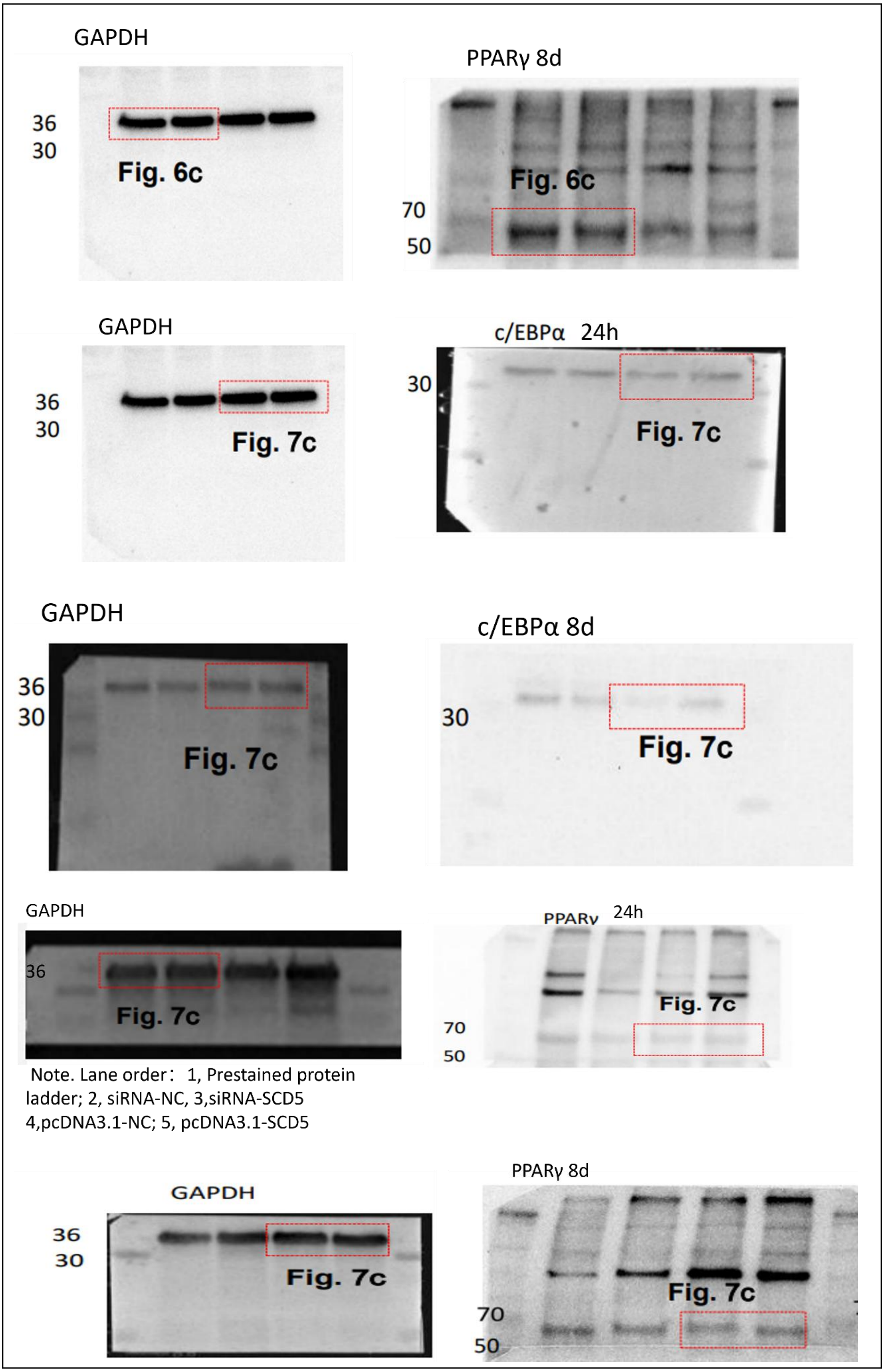

Note. Lane order： 1, Prestained protein ladder; 2, siRNA-NC, 3,siRNA-SCD5 4,pcDNA3.1-NC; 5, pcDNA3.1-SCD5

Supplementary Figure S4. Full-length blot for β-catenin, p-β-catenin, and WNT5B (corresponding to Fig. 9c).

Lane 1: Prestained protein ladder; Lane 2: siRNA-NC; Lane 3: siRNA-WNT5B.

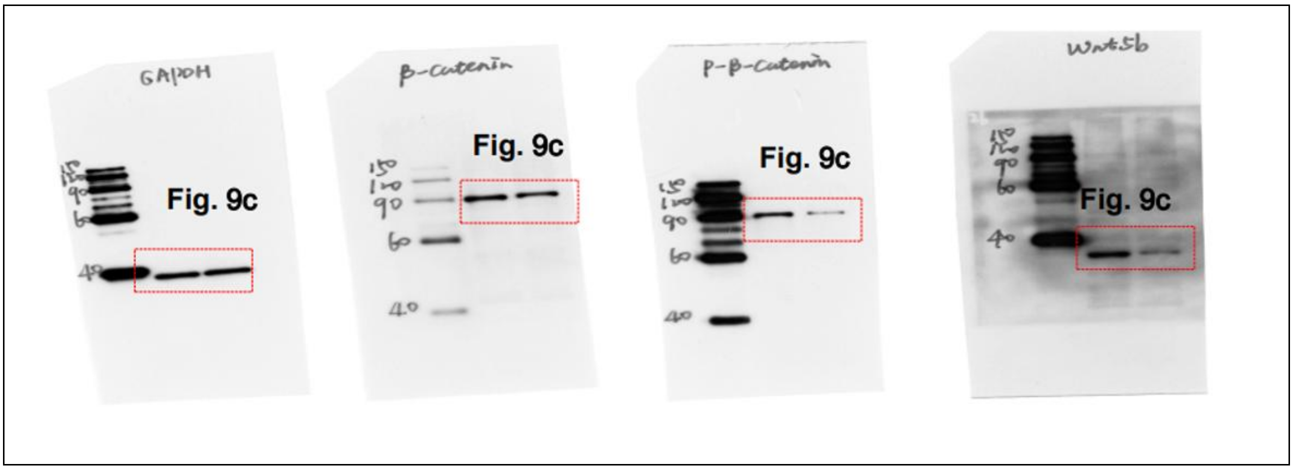

Supplement: Supplementary file 1 [file animals-15-03547-s001.zip › animals-3985375-supplementary.pdf]
